# Comparisons of outcomes in stroke subtypes after intravenous thrombolysis

**DOI:** 10.1186/s40064-016-1666-y

**Published:** 2016-01-20

**Authors:** Yi-Ting Pan, Jiann-Der Lee, Ya-Hui Lin, Ying-Chih Huang, Hsu-Huei Weng, Meng Lee, Chih-Ying Wu, Huan-Lin Hsu, Hsin-Ta Yang, Chia-Yu Hsu, Tsong-Hai Lee, Shan-Jin Liu, Tsung-Yi Peng, Chia-Wei Liou, Ku-Chou Chang, Yen-Chu Huang

**Affiliations:** Department of Neurology, Chang Gung Memorial Hospital, Chang-Gung University College of Medicine, 6 West Chia-Pu Road, Putz City, Chiayi County Taiwan; Department of Diagnostic Radiology, Chang Gung Memorial Hospital, Chang-Gung University College of Medicine, Putz City, Chiayi County Taiwan; Department of Neurology, Stroke Center, Chang Gung Memorial Hospital Linkou Medical Center, Chang Gung University College of Medicine, Taoyuan County, Taiwan; Department of Neurology, Stroke Center, Chang Gung Memorial Hospital, Chang Gung University College of Medicine, Keelung City, Taiwan; Department of Neurology, Stroke Center, Chang Gung Memorial Hospital Kaohsiung Medical Center, Chang Gung University College of Medicine, Kaohsiung City, Taiwan

**Keywords:** Acute ischemic stroke, Intravenous thrombolysis, Stroke subtype

## Abstract

The purpose of this study was to analyze the outcomes and complications between stroke subtypes after intravenous thrombolysis. A total of 471 patients with acute ischemic stroke after intravenous thrombolysis from January 2007 to April 2014 were enrolled and classified according to the Trial of Org 10172 in Acute Stroke Treatment. A multivariate logistic regression model was used to evaluate the outcomes and complications among stroke subtypes after adjusting for baseline variables. Of the 471 patients, 117 (25.1 %) had large-artery atherosclerosis (LAA), 148 (31.8 %) had cardioembolism (CE), 82 (17.6 %) had small vessel disease (SVD), 119 (25.5 %) had undetermined etiology, and 5 (1.1 %) had other determined etiology. The patients with SVD had the mildest initial stroke severity and highest ratio of good and favorable outcomes, whereas those with CE had a higher rate of symptomatic intracranial hemorrhage (sICH) than those with SVD. After adjusting for confounding factors, the ratio of favorable outcome in the patients with SVD stroke was higher than in those with LAA. SVD was associated with a significantly lower rate of any hemorrhage compared to other stroke subtypes, whereas there were no differences in sICH or mortality between stroke subtypes. A lower initial National Institutes of Health Stroke Scale score was associated with good and favorable outcomes, and lower rates of sICH and mortality. The patients with SVD after intravenous thrombolysis had better outcomes and a lower rate of hemorrhage even after adjusting for confounding factors. Stroke severity was an independent factor associated with better functional outcomes, sICH and mortality.

## Background

Thrombolysis treatment with intravenous recombinant human tissue plasminogen (tPA) is currently the only widely approved medical treatment for acute ischemic stroke. In selected patients, it has been found to improve functional recovery with an acceptable risk (The National Institute of Neurological Disorders and Stroke rt-PA Stroke Study Group 1995; Hacke et al. 1995; Wardlaw et al. 2012). According to the etiology, ischemic stroke can be classified into several subtypes which may be related to a different prognosis (Ois et al. 2013), most likely due to differences in the mechanism.

Although tPA has been shown to be effective in previous reports, few studies have evaluated its efficacy and complications between ischemic stroke subtypes after thrombolysis. In the National Institute of Neurological Disorders and Stroke (NINDS) and European Cooperative Acute Stroke Study (ECASS) studies (The National Institute of Neurological Disorders and Stroke rt-PA Stroke Study Group. 1995; Hacke et al. 1995), the use of tPA was not restricted to patients with small vessel disease (SVD), and whether or not thrombolysis is beneficial for these patients is under debate. In addition, the efficacy of tPA has been reported to be related to the size, composition and origin of the thrombus (Molina et al. 2004). In addition, although the fresh blood clots originating mainly from cardioembolism (CE) stroke can be more easily dissolved by thrombolytic agents than old blood clots, patients with CE stroke are generally older with more severe strokes. These factors may mean that there is no difference in outcome between patients with CE and large-artery atherosclerosis (LAA) stroke (Fuentes et al. 2012; Mustanoja et al. 2011). The purpose of this study was to analyze the outcomes and complications between stroke subtypes after intravenous tPA treatment.

## Subjects and methods

We retrospectively analyzed the clinical data of patients with acute ischemic stroke who received intravenous tPA treatment at a Chang Gung Memorial Hospital (there are four branches at Linkou, Kaohsiung, Keelung, and Chiayi) from January 2007 to April 2014. The clinical data of these acute stroke patients were prospectively registered in the Chang Gung Stroke registry databank. We identified 499 patients who received intravenous tPA treatment, 28 of whom with a baseline modified Rankin scale (mRS) score >1 were excluded. A total of 471 patients were analyzed and classified according to the Trial of Org 10172 in Acute Stroke Treatment (TOAST), including LAA, CE, SVD, undetermined etiology (UnD) and other determined etiology. The outcomes after intravenous thrombolysis included mRS on discharge from the hospital, symptomatic intracranial hemorrhage (sICH), any hemorrhage, and mortality. A good outcome was defined as a mRS score ≤2, and a favorable outcome was defined as a mRS score ≤1. Symptomatic ICH was defined according to the ECASS criteria (Hacke et al. 1995). The study was approved by the Institutional Review Board of Chang Gung Memorial Hospital (ethical license No.: 102-4340B).

### Statistical analysis

All statistical analyses were performed using the Statistical Program for Social Sciences (SPSS) statistical software (version 18). Continuous variables were expressed as mean ± standard deviation or median and interquartile range. Variables were compared using the Kruskal–Wallis test, and a Bonferroni-corrected Mann–Whitney U test was used to identify which two-by-two comparisons were significantly different. Rates of different categories among the groups were compared using the Chi square test. A multivariate logistic regression model was used to evaluate the outcomes after adjusting for baseline variables with a *p* value <0.1 in univariate analysis. All statistical tests were two-tailed, and a *p* value of <0.05 was considered to be statistically significant.

## Results

Of the 471 patients who received intravenous tPA treatment, 82 (17.6 %) had SVD, 148 (31.8 %) had CE, 117 (25.1 %) had LAA, 119 (25.5 %) had UnD, and 5 (1.1 %) had other determined etiology. The other determined etiology was not analyzed because of the small number of patients. The demographic data of these patients are shown in Table 1. The patients with SVD had the mildest stroke severity (median NIHSS = 7), while those with CE had the worst stroke severity (median NIHSS = 15). The initial NIHSS in the patients with SVD was significantly lower than in those with CE (*p* < 0.001) and LAA (*p* < 0.001). The patients with CE were the oldest among all stroke subtypes, and it was significantly different compared to those with LAA (*p* < 0.001). The patients with LAA were predominantly male, and there were significant differences compared to those with CE (*p* < 0.001) and SVD (*p* = 0.004). Because atrial fibrillation was used to determine the diagnosis of CE stroke, the ratio was higher in the patients with CE stroke than in those with other stroke subtypes (CE vs. LAA, *p* < 0.001; CE vs. SVD, *p* < 0.001; CE vs. UnD, *p* < 0.001). Congestive heart failure was more prevalent in the patients with CE than in those with other stroke subtypes, whereas hyperlipidemia was less prevalent in the patients with CE than in those with LAA (*p* = 0.001) and SVD (*p* = 0.003). There were no differences between the stroke subtypes in other stroke risk factors, time to the initiation of tPA treatment or initial blood pressure.

**Table 1:** The demographic data of patients with IV tPA treatment

	SVD	CE	LAA	UnD	*p*
N	82	148	117	119	
Age (mean ± SD)	64.5 ± 11.3	68.3 ± 11.5	61.5 ± 13.2	64.9 ± 13.1	<0.001
Sex (F/M)	34/48	74/74	26/91	39/80	<0.001
Atrial fibrillation (%)	0 (0 %)	136 (91.9 %)	2 (1.7 %)	43 (36.1 %)	<0.001
Diabetes mellitus (%)	30 (36.6 %)	36 (24.3 %)	30 (25.6 %)	35 (29.4 %)	0.220
Hypertension (%)	68 (82.9 %)	110 (74.3 %)	89 (76.1 %)	91 (76.5 %)	0.516
Hyperlipidemia (%)	46 (56.1 %)	49 (33.1 %)	62 (53.0 %)	58 (48.7 %)	<0.001
Coronary artery disease (%)	3 (3.7 %)	23 (15.5 %)	13 (11.1 %)	12 (10.1 %)	0.051
Congestive heart failure (%)	1 (1.2 %)	19 (12.8 %)	2 (1.7 %)	3 (2.5 %)	<0.001
Old stroke (%)	16 (19.5 %)	18 (12.2 %)	17 (14.5 %)	19 (16.0 %)	0.503
Smoking (%)	27 (32.9 %)	34 (23.0 %)	40 (34.2 %)	39 (32.8 %)	0.214
Prior antiplatelet treatment (%)	13 (15.9 %)	47 (31.8 %)	13 (11.1 %)	26 (21.8 %)	<0.001
Prior anticoagulant treatment (%)	1 (1.2 %)	10 (6.8 %)	0 (0 %)	3 (2.5 %)	0.008
Systolic BP (mmHg)	156.1 ± 21.7	148.4 ± 27.8	152.0 ± 25.0	148.1 ± 21.8	0.064
Diastolic BP (mmHg)	86.6 ± 14.5	86.3 ± 18.1	85.6 ± 16.0	84.0 ± 13.7	0.814
Onset to tPA time (min)	135.7 ± 46.2	133.5 ± 71.6	135.3 ± 42.2	132.5 ± 42.0	0.647
NIHSS baseline, median (IQR)	7 (6–10)	15 (9–20)	12 (8–18)	11 (7–17)	<0.001
NIHSS 2 h, median (IQR)	5 (4–8.25)	13 (7–18)	11 (7–16)	9 (5–16)	<0.001
NIHSS discharge, median (IQR)	3 (1–6.25)	7 (2–15)	7 (3–13)	6 (2–11)	<0.001
MRS admission, median (IQR)	4 (2–4)	4 (4–5)	4 (4–5)	4 (3–5)	<0.001
MRS discharge, median (IQR)	1.5 (1–4)	4 (1–5)	4 (1.5–5)	3 (1–5)	<0.001
MRS at discharge ≤1	41 (50.0 %)	40 (27.0 %)	29 (24.8 %)	36 (30.3 %)	0.001
MRS at discharge ≤2	49 (59.8 %)	43 (29.1 %)	38 (32.5 %)	45 (37.8 %)	<0.001
Symptomatic ICH	1 (1.2 %)	17 (11.5 %)	5 (4.3 %)	7 (5.9 %)	0.012
Any ICH	2 (2.4 %)	50 (33.8 %)	28 (23.9 %)	19 (16.0 %)	<0.001
Mortality	0 (0 %)	15 (10.1 %)	5 (4.3 %)	6 (5.0 %)	0.011

There was a significant difference between stroke subtypes in favorable and good outcomes (*p* < 0.001). The patients with SVD had the highest ratio of good and favorable outcomes compared to the other stroke subtypes (good outcome: SVD–CE *p* < 0.001, SVD–LAA *p* < 0.001, SVD–UnD *p* = 0.001; favorable outcome: SVD–CE *p* < 0.001, SVD–LAA *p* < 0.001, SVD–UnD *p* = 0.002). The higher ratio of favorable outcome between SVD and LAA was still significantly different after adjusting for confounding factors (Table 2). Furthermore, a lower initial NIHSS was associated with favorable and good outcomes, and younger age was associated with favorable outcome (Table 2).

**Table 2:** Multivariate logistic regression model for outcomes

	Favorable outcome		Good outcome		Symptomatic ICH		Any hemorrhage		Mortality	
	Odds ratio	*p*	Odds ratio	*p*	Odds ratio	*p*	Odds ratio	*p*	Odds ratio	*p*
TOAST		0.037		0.080		0.503		0.003		
LAA versus SVD	0.39 (0.18–0.69)	0.005	0.46 (0.23–0.83)	0.011	2.50 (0.27–23.7)	0.423	11.03 (2.51–48.6)	0.002		
CE versus SVD	0.69 (0.28–1.70)	0.415	0.73 (0.30–1.78)	0.482	5.60 (0.54–58.5)	0.150	12.26 (2.43–61.9)	0.003		
UnD versus SVD	0.53 (0.28–1.03)	0.059	0.60 (0.31–1.14)	0.118	3.84 (0.42–35.2)	0.234	6.07 (1.31–28.1)	0.021		
CE versus LAA	1.76 (0.72–4.29)	0.215	1.67 (0.70–3.96)	0.248	2.24 (0.49–10.2)	0.297	1.11 (0.46–2.72)	0.816		
Initial NIHSS	0.92 (0.89–0.96)	<0.001	0.91 (0.88–0.95)	<0.001	1.08 (1.02–1.14)	0.007	1.03 (0.995–1.07)	0.091	1.13 (1.07–1.20)	<0.001
Age	0.98 (0.96–0.999)	0.043	0.99 (0.97–1.00)	0.088	1.02 (0.98–1.05)	0.330			1.04 (0.99–1.08)	0.111
Sex (male)	1.54 (0.92–2.57)	0.098	1.31 (0.80–2.14)	0.281	0.50 (0.23–1.10)	0.083			0.47 (0.19–1.16)	0.101
Atrial fibrillation	0.94 (0.44–2.00)	0.862	0.68 (0.32–1.42)	0.300	0.93 (0.28–3.12)	0.906	1.29 (0.59–2.81)	0.526	1.33 (0.50–3.55)	0.572
Hyperlipidemia					0.69 (0.30–1.60)	0.385			0.13 (0.03–0.59)	0.008
Smoking	1.36 (0.81–2.27)	0.247	1.39 (0.84–2.31)	0.199						
Prior anticoagulant treatment							1.92 (0.60–6.09)	0.270	3.78 (0.71–20.1)	0.119
Congestive heart failure									1.31 (0.31–5.51)	0.714

Baseline variables with a *p* value <0.1 in univariate analysis were included in multivariate logistic regression models for each outcome. Favorable and good outcomes: TOAST classification, initial NIHSS, age, sex, atrial fibrillation and smoking; symptomatic ICH: TOAST classification, initial NIHSS, age, sex, atrial fibrillation and hyperlipidemia; any hemorrhage: TOAST classification, initial NIHSS, atrial fibrillation and prior anticoagulant treatment; mortality: initial NIHSS, age, sex, atrial fibrillation, hyperlipidemia, prior anticoagulant treatment and congestive heart failure

The ratio of any hemorrhage and sICH was highest in those with CE and lowest in those with SVD (Table 1). The ratio of sICH was significantly higher in those with CE than in those with SVD (*p* = 0.005), whereas the ratio of any hemorrhage in those with SVD was significantly lower than in those with any other stroke subtype. More patients with CE received antiplatelet or anticoagulant treatment than those with LAA (*p* < 0.001 and *p* = 0.004). However, after adjusting for confounding factors, sICH was not significantly different between the stroke subtypes, although the patients with SVD still had a significantly lower ratio of any hemorrhage compared to those with any other stroke subtype. Initial NIHSS was associated with sICH independently of the underlying factors (Table 2). A higher NIHSS and absence of hyperlipidemia were significantly associated with a higher mortality rate (Table 2).

## Discussion

Our findings show that SVD after intravenous thrombolytic treatment was associated with a better outcome than LAA and a lower intracranial hemorrhage rate than any other stroke subtype, even after adjusting for confounding factors. The patients with CE had a higher rate of sICH than those with SVD, however the difference disappeared after adjusting for initial stroke severity. Initial stroke severity was an independent factor for favorable and good outcomes, sICH, and mortality in the patients treated with thrombolytic treatment.

These results support that the patients with SVD who were treated with tPA had better clinical outcomes, even after adjusting for baseline confounding factors. However, previous studies have reported no differences between stroke subtypes with or without adjusting for confounding factors (Fuentes et al. 2012; Cocho et al. 2006; Hsia et al. 2003). Although the ECASS and NINDS trials showed the efficacy of intravenous tPA in all patients regardless of stroke subtype (The National Institute of Neurological Disorders and Stroke rt-PA Stroke Study Group 1995; Hacke et al. 1995), a subsequent study proposed that the benefits for patients with SVD may be attributable to a younger age and milder stroke severity (Hsia et al. 2003; Fluri et al. 2010). Moreover, SVD is not thought to be susceptible to reperfusion treatment (Cocho et al. 2006) due to the lack of existing lysible clots and penumbra (Hwang et al. 2008). However, several studies have reported that patients with lacunar stroke may have better outcomes after thrombolysis (Lahoti et al. 2014; Shobha et al. 2013). In the current study and a study by Mustanoja et al. (2011), SVD was an independent factor for a good prognosis. In addition, some pathophysiology of lacunar infarcts has been attributed to atheroma or embolism rather than lipohyalinosis alone (Wardlaw et al. 2013; Jeong et al. 2014), and perfusion defects have been observed in lacunar infarcts on magnetic resonance perfusion scans (Poppe et al. 2009; Huang et al. 2014), supporting the micro-penumbra hypothesis. Thus, thrombolytic treatment may be beneficial for these patients.

Increasing evidence indicates that the natural course of patients with minor ischemic stroke is not as good as previously thought (Guerrero and Savitz 2013). Early neurological deterioration (END) has been reported to occur in 20–30 % patients with SVD despite minor stroke symptoms (Hwang et al. 2008; Audebert et al. 2004), and this may lead to functional disability (lower Barthel Index score) (Audebert et al. 2004). A recent study also demonstrated that END in patients with acute lacunar infarction is associated with the transformation of non-core hypoperfused areas into infarctions (Huang et al. 2014), suggesting that a lysible clot may prevent END. In our study, 15 of 82 patients with SVD had minor stroke severity with a NIHSS ≤5. Our results indicate that patients with SVD, and usually those with minor stroke symptoms, had better outcomes and a lower intracranial hemorrhage rate. Therefore, intravenous tPA treatment may be beneficial for patients with SVD even if they only have minor symptoms.

SVD was associated with a low incidence of symptomatic ICH in the current study (1.6 %) and other studies (0 and 4.6 %) (Hsia et al. 2003; Fluri et al. 2010). In contrast, SVD was associated with a higher prevalence of microbleeds, ranging from 45.7 to 62.1 %, compared to the other stroke subtypes (Cordonnier et al. 2007; Schonewille et al. 2005; Naka et al. 2004; Kato et al. 2002). Since microbleeds are highly associated with lobar ICH from cerebral amyloid angiopathy, they are presumably associated with an increased risk of bleeding. However, the symptomatic ICH rate in the patients with SVD after thrombolysis was not higher than expected based on the high percentage of microbleeds. A possible reason for this is that microbleeds are markers of SVD representing underlying pathologies of hypertensive vasculopathy or amyloid angiopathy rather than real-time bleeding. Moreover, the microbleeds in SVD are more likely due to hypertensive vasculopathy, which is less associated with ICH (Inzitari et al. 1990; Smith et al. 2010). Therefore, further studies are needed to clarify whether microbleeds increase the rate of hemorrhage after thrombolysis.

Our results revealed that the patients with CE stroke had a higher rate of sICH than those with SVD, however the difference disappeared after adjusting for confounding factors. The higher rate of sICH may be due to the greater severity of stroke in the patients with CE stroke due to the large infarct areas. In addition, CE stroke is related to a larger core infarct and smaller penumbra (Kim et al. 2009), and a larger core infarct has been reported to be associated with sICH, especially in patients with early reperfusion after thrombolytic therapy (Albers et al. 2006). Therefore, the rate of sICH should be higher in patients with CE, especially in those with a larger infarct and early recanalization.

Although the fresh blood clots in CE stroke should be more sensitive to thrombolytic agents, we found no significant difference in favorable or good outcomes between the patients with CE and LAA strokes after adjusting for underlying confounding factors. A possible reason for this is that the greater disease severity in CE may mean that the blood clot in the occluded vessel is bigger and more difficult to dissolve. Another possible reason is that the penumbra to core infarct ratio is usually lower in CE than in LAA (Kim et al. 2009), and that the effect of intravenous tPA treatment may be limited despite recanalization. Finally, CE was associated with a higher rate of sICH compared to LAA stroke (11.5 vs. 4.3 %, *p* = 0.035), and this may be related to the poorer outcomes.

A potential limitation to the current study is that we did not compare the outcomes of SVD directly between patients receiving thrombolysis and a placebo. However, a placebo-controlled study comparing intravenous tPA outcomes may be not ethically acceptable. Moreover, mRS was evaluated at discharge rather than at 3 months. Although the mRS at discharge has been reported to be highly correlated with the final functional outcome (Banks and Marotta 2007), there may be some discrepancies concerning the actual recovery after stroke. In addition, this study is limited by the small number of cases, which also consists of heterogeneous data.

## Conclusion

SVD after intravenous thrombolysis was associated with favorable outcomes and fewer hemorrhages even after adjusting for confounding factors. Initial stroke severity was an independent factor for a better outcome, sICH, any hemorrhage, and mortality rate regardless of the stroke subtype.
